# The description of a method for accurately estimating creatinine clearance in acute kidney injury

**DOI:** 10.1016/j.mbs.2016.02.010

**Published:** 2016-03-10

**Authors:** John Mellas

**Affiliations:** Department of Internal Medicine, St Mary’s Health Center, 6400 Clayton Road, St Louis, Missouri, 63017, United States

**Keywords:** Acute kidney injury, Glomerular filtration rate, Creatinine clearance, Creatinine production, Creatinine excretion

## Abstract

**Background:**

Acute kidney injury (AKI) is a common and serious condition encountered in hospitalized patients. The severity of kidney injury is defined by the RIFLE, AKIN, and KDIGO criteria which attempt to establish the degree of renal impairment. The KDIGO guidelines state that the creatinine clearance should be measured whenever possible in AKI and that the serum creatinine concentration and creatinine clearance remain the best clinical indicators of renal function. Neither the RIFLE, AKIN, nor KDIGO criteria estimate actual creatinine clearance. Furthermore there are no accepted methods for accurately estimating creatinine clearance (K) in AKI.

**Study design:**

The present study describes a unique method for estimating K in AKI using urine creatinine excretion over an established time interval (E), an estimate of creatinine production over the same time interval (P), and the estimated static glomerular filtration rate (sGFR), at time zero, utilizing the CKD-EPI formula. Using these variables estimated creatinine clearance (Ke) = E/P ∗ sGFR.

**Setting and participants:**

The method was tested for validity using simulated patients where actual creatinine clearance (Ka) was compared to Ke in several patients, both male and female, and of various ages, body weights, and degrees of renal impairment. These measurements were made at several serum creatinine concentrations in an attempt to determine the accuracy of this method in the non-steady state. In addition E/P and Ke was calculated in hospitalized patients, with AKI, and seen in nephrology consultation by the author. In these patients the accuracy of the method was determined by looking at the following metrics; E/P > 1, E/P < 1, E = P in an attempt to predict progressive azotemia, recovering azotemia, or stabilization in the level of azotemia respectively. In addition it was determined whether Ke < 10 ml/min agreed with Ka and whether patients with AKI on renal replacement therapy could safely terminate dialysis if Ke was greater than 5 ml/min.

**Outcomes and results:**

In the simulated patients there were 96 measurements in six different patients where Ka was compared to Ke. The estimated proportion of Ke within 30% of Ka was 0.907 with 95% exact binomial proportion confidence limits. The predictive accuracy of E/P in the study patients was also reported as a proportion and the associated 95% confidence limits: 0.848 (0.800, 0.896) for E/P < 1; 0.939 (0.904, 0.974) for E/P > 1 and 0.907 (0.841, 0.973) for 0.9 < E/P < 1.1. Ke < 10 ml/min correlated very well with Ka, while Ke > 5 ml/min accurately predicted the ability to terminate renal replacement therapy in AKI.

**Limitations:**

Include the need to measure urine volume accurately. Furthermore the precision of the method requires accurate estimates of sGFR, while a reasonable measure of P is crucial to estimating Ke.

**Conclusions:**

The present study provides the practitioner with a new tool to estimate real time K in AKI with enough precision to predict the severity of the renal injury, including progression, stabilization, or improvement in azotemia. It is the author’s belief that this simple method improves on RIFLE, AKIN, and KDIGO for estimating the degree of renal impairment in AKI and allows a more accurate estimate of K in AKI.

## 1. Introduction

Acute kidney injury (AKI) is a common and serious medical condition encountered in hospitalized patients. When present it contributes significantly to morbidity, mortality, and overall health care costs due to the derangements in fluid, electrolyte, and acid base balance which further complicate the course of an illness and increase the challenge of managing these often devastating metabolic consequences [1–13].

The most recent classifications estimate the degree of renal impairment using the Risk, Injury, Failure, Loss, End stage renal disease (RIFLE), Acute Kidney Injury Network (AKIN) and The Kidney Disease Improving Global Outcomes (KDIGO) criteria. These methods attempt to estimate the degree of kidney injury using a measure of absolute or relative rise in serum creatinine concentration or determining the duration of oliguria [2,9–12]. Shortcomings to these criteria include the fact that neither method estimates actual glomerular filtration rate (GFR) or creatinine clearance (K) and hence infer the extent of renal injury. Furthermore, by relying on serum creatinine concentration (C) and changes in C (dC/dt), there are inherent errors related to different creatinine kinetics which vary between patients, based on muscle mass, making direct correlations between patients unreliable. Additionally, there are situations where significant falls in GFR are not detected due to a delay in the rise in C. There are also dilution effects of parenteral solutions on C and dC/dt. It is for these reasons that AKIN, RIFLE, KDIGO or other methods which rely on C are not reliable metrics for accurately measuring GFR or K in AKI. Finally, the estimate of renal injury is made retrospectively and does not allow one to predict the course of the renal impairment prospectively [14–21].

Regardless of the method used to establish the diagnosis of AKI, KDIGO states that the serum creatinine concentration (C) and creatinine clearance (K) remain the single best markers of kidney function and should be measured whenever possible [2,22–25]. Formulas to measure K in AKI have been described [26–29] and are equations based on creatinine mass balance in the non-steady state (S appendix). Although mathematically accurate they are not ideal for clinical use due to the fact they require an accurate value for creatinine distribution volume (Vd), as well as a measure of changing creatinine concentration (dC/dt). These values are difficult to measure precisely and are prone to large errors related to parenteral fluid administration and dilution of the value for Vd and dC/dt, rendering these methods impractical for clinical use [20,21].

This paper describes a method relying on principals of creatinine mass balance and K estimation using the urine creatinine excretion (E) as opposed to serum creatinine (C). This method is more accurate than those relying on serum creatinine in that urine creatinine concentration (U) and urine volume (V) are measured directly and accurately, and not estimated, while the term static K (Ks) is introduced and represents the creatinine clearance as determined by CKD-EPI, at any time zero in the course of AKI.

## 2. Methods

### 2.1. Description of formula

In the steady state creatinine production (P) is equal to renal creatinine excretion (E). In such cases the serum creatinine concentration is constant with K = U∗V/C, where K is inversely proportional to C [16,24,29,30]. Furthermore the steady state allows one to estimate GFR by using estimating formulas such as MDRD or CKD-EPI. From this one can appreciate that when E is less than P, C will rise and the patient is in AKI. When E is greater than P, C is falling and the patient is recovering from AKI. When E is zero, K is zero. Finally, when E = P, a steady state is reached and may represent normalization of renal function or a plateau has been reached in the course of AKI where C is stable [21].

**Fig. 1.**
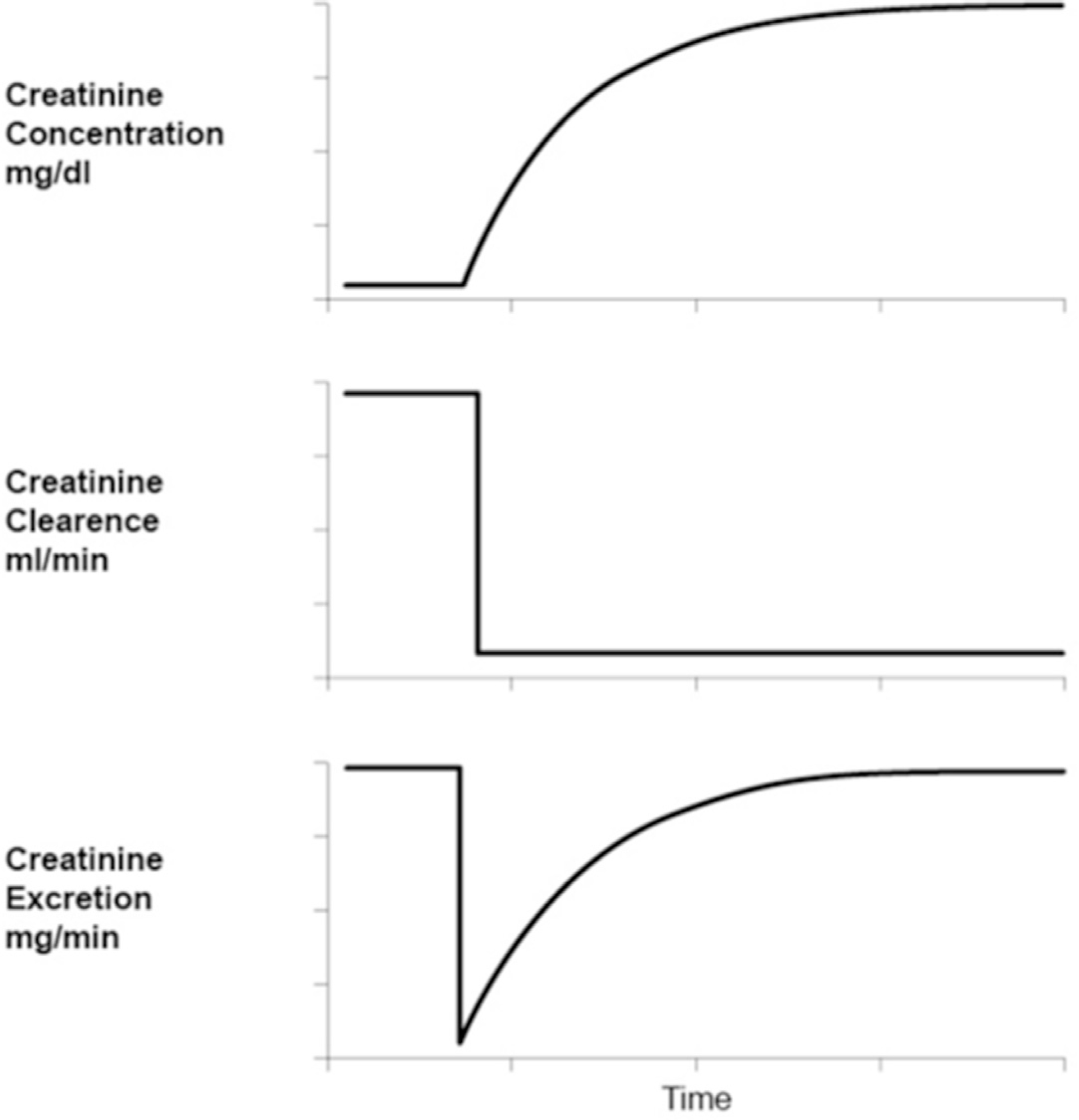
Creatinine kinetics in AKI. Intuitively, one can appreciate that as you move from left to right along the X-axis, the ratio of creatinine excretion over production (E/P) rises at any time interval, while the estimated static K (Ks) at the start of that time interval falls. Hence, a rising E/P multiplied by a falling Ks at any time (t) is a constant number and equals the actual K where K = Ks*E/P. In essence, E/P represents a “correction factor” for the Ks at any time (t). At the plateau phase, to the far right of the graph, the creatinine no longer rises, E/P = 1, thus 1 multiplied by Ke = Ka.

**Fig. 2.**
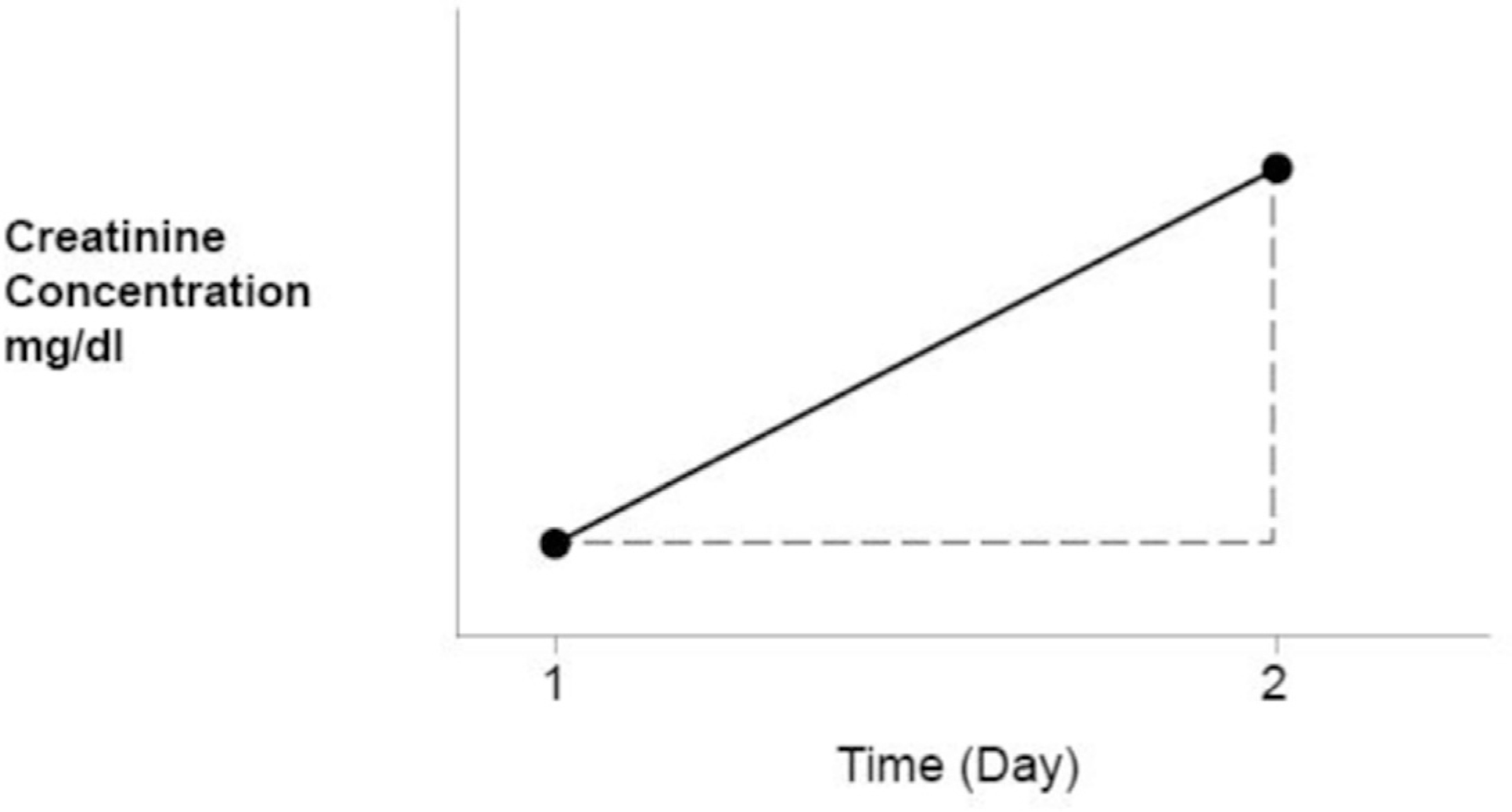
Creatinine kinetics in AKI. A_n_ = Amount of creatinine retained in the serum per day A_n_ = Vd ∗ (C2−C1) = P−E, therefore, E = P−A_n_ E/P = 1−A_n_/P. When A_n_ = P, E/P = 0. When A_n_ = 0, E = P and E/P = 1. For each point, A_n_ between 0 and 1 actual GFR is proportional to E/P.

Fig. 1 shows the simulation of a patient with AKI with a sudden drop in K from 100 ml/min to 10 ml/min. This is associated with an abrupt fall in E followed by a steady rise until E = P and a new steady state has been reached reflecting the actual K of 10 ml/min. The slope of dC/dt is positive reflecting the net accumulation of creatinine in the serum. At each time interval, the area An represents the net serum accumulation of creatinine, which is equal to Vd ∗ (C2 – C1) where Vd is the distribution volume for creatinine (Fig. 2). Moving from left to right across the x axis in Fig. 1, as each area An falls, there is a reciprocal rise in E, while the ratio E/P rises until finally C peaks at the level that corresponds to the actual K, which in this example equals 10 ml/min. At this point in time a new steady state has been reached, where An = 0, as C is no longer rising, E = P, and K can be estimated directly or measured.

In Fig. 2 one sees that E = P – An (where An = Vd ∗ (C2 – C1). Rearranging we find that E/P = 1 – An/P. When An is large, E/P is small and GFR is low. When An is small, E/P approaches one and a plateau is being reached. When An is zero, E = P. This represents the plateau and is the only time when K can be estimated or measured in AKI by standard methods.

From this one can appreciate that when: E/P < 1, the patient is in AKI, E/P > 1 the patient is in a recovery pattern, E/P = 1 a plateau has been reached and GFR can be estimated or measured, and finally when E/P = 0, K is zero.

Referring again to Fig. 1 the method can be developed further. At any point in the graph between the onset of renal injury and the plateau there is progressive fall in estimated K by CKD-EPI or MDRD corresponding to each changing value for C. Each measure for K would only be valid if E was equal to P from that corresponding time going forward. The level for estimated K at any point in time is called static K (Ks) and represents the actual K if indeed one was in a steady state. With each corresponding time interval, from onset of injury to plateau, there is a progressive fall in the value for Ks accompanied by a proportional rise in E/P so that, at any time interval, the product of Ks at time 0 and E/P at time 0 to time 1 is a constant and is in fact equal to Ke.

The final formula states that; Ke = Ks ∗ E/P at any time interval t0 to t1, where Ks equals K at t0 by CKD-EPI, E is measured directly as U∗V over the same time interval, and P is estimated by the following formulas as described by Bjornsson; P (male) = (27−.173 ∗ age) ∗ weight in kg, and P (female) = (25−.175 ∗ age) ∗ weight in kg over 24 h [11]. Where weight is lean body weight and P falls by 2% for each hospital day [21].

### 2.2. Simulated patients

To prove that the formula Ke = Ks∗E/P was valid involved a more detailed analysis of the mathematics of creatinine kinetics in AKI. Beginning with the basic formula for creatinine clearance in AKI, where dC/dt = P/V-C∗K/V. The integral of this formula solves for C with respect to t, where C(t) = P/K + [C(0)−P/K]∗ê−Kt/V [16,28 and Fig. 1].

Six different patients were simulated (Tables 1–6) including variables of baseline renal function and renal function after AKI (Ka), body weight, age, and gender. V was estimated as 60% of body weight corresponding to total body water as an estimate of creatinine distribution volume [29], P was estimated by the method described above, and C, at time zero, was directly calculated from the equation K = P/C in the steady state. For each patient V∗dC/dt was calculated at several time intervals, and was equal to V [C(t) – C(0)] divided by the time interval, which represents the accumulation of creatinine over time in AKI. Knowing that E = P–V∗dC/dt, E was calculated directly. The values of Ks (by CKD-EPI) at any time (t) multiplied by E/P at several time intervals (Ke) in each of the modeled patients were compared with Ka in each patient. Comparisons were made between 4 h and 24 h time intervals. The calculations were repeated where actual P was over estimated by 20% and V was 50% of body weight as compared to 60%. The values for Ke were then compared with Ka.

### 2.3. Study patients

These were patients seen by the author in nephrology consultation between February 2010 and March 2015 after having met the inclusion criteria. The use of data in the study patients was approved by the SSM Health Institutional Review Board Protocol Number 13–02-0293. The committee ruled that informed consent was not required. All requests for consultation were for evaluation of AKI due to the onset of azotemia or oliguria. Inclusion criteria included a diagnosis of AKI based on a rising serum creatinine concentration or a period of oliguria defined as a urine output less than 20 ml/h for eight consecutive hours. An indwelling bladder catheter was required for the accurate measure of V. Measurements of U, daily C, with Ks estimated by CKD-EPI were recorded. A total of 366 patients were studied with 586 measurements made for Ke. The study patients were divided by the following categories:

1. E/P < 1, predicting AKI based on any rise in C from t0 within 24 h.
2. E/P > 1, predicting AKI recovery based on a falling C from t0 within 24 h.
3. E/P > 0.9 and < 1.1 predicting renal function stabilization with C ± 10% from C at t0 for a period of 24–48 h.
4. Ke < 10 ml/min predicting the need for eventual renal replacement therapy (RRT), after allowing a period of 24 h for intensive resuscitation by standard methods, or a peak steady state C corresponding to K < 10 ml/min by CKD-EPI with RRT not undertaken due to patient refusal or other clinical factors.
5. Ke > 5 ml/min predicting the withdrawal of RRT in patients with AKI who had RRT initiated based on clinical judgment.

### 2.4. Statistical analysis

Statistical analysis was performed on data from the six simulated patients. The values for Ke (E/P∗Ks) were compared to their true values Ka (gold standard). The estimated proportion of Ke within 20% of Ka was 0.849 with 95% exact binomial proportion confidence limits (0.773, 0.925). The estimated proportion of Ke within 30% of Ka was 0.907 with 95% exact binomial proportion confidence limits (0.825, 0.959). The predictive accuracy of E/P was also reported as a proportion and the associated 95% confidence limits: 0.848 (0.800, 0.896) for E/P < 1; 0.939 (0.904, 0.974) for E/P > 1 and 0.907 (0.841, 0.973) for 0.9 < E/P < 1.1. All statistical analyses were conducted with SAS 9.4 (SAS Institute, Cary, NC).

## 3. Results

### 3.1. Simulated patients

Simulated patients were evaluated for predictive value for each measurement made where Ka was compared to Ke in the modeled patients and as well as when actual P fell by 20% below estimated P and V was 50% body weight instead of 60%. These measurements were made for 4 and 24 h urine collections. K in AKI was divided into deciles of 10 ml/min, between 0 and 100 ml/min to determine whether Ke fell in any particular decile as predicted by the equation Ke = Ks∗E/P. Each patient in the 4 h urine collection had a Ke that fell within a range of 10 ml/min compared to Ka. Most estimates for Ke differed from Ka by less than 5 ml/min.

When Ke was compared to Ka for an actual P which was 20% less than estimated P and V which was 50% instead of 60% body weight, the results were similar with all values of Ke less than 10 ml/min different from Ka in the 4 h urine measurements, with most differences less than 5 ml/min.

### 3.2. Study patients

A total of 366 patients were studied between February 2010 and March 2015 with 586 measurements for K.

E/P < 1; There were 184 of 217 measurements which were followed by a rising C for a predictive accuracy of 85%.

E/P > 1; There were 169 of 180 measurements which were followed by a falling C for a predictive accuracy of 94%.

.9 < E/P < 1.1; There were 68 of 75 measurements followed by a stable C over 24–48 h for a predictive accuracy of 91%.

Ke < 10 ml/min; There were 68 of 74 measurements followed by the need for renal replacement therapy or a peak C which corresponded to a Ka of < 10 ml/min for a predictive accuracy of 92%.

Ke > 5 ml/min; There were 40 of 40 measurements which allowed renal replacement therapy, initiated for the treatment of AKI, to be safely terminated with a predictive accuracy of 100%.

## 4. Discussion

AKI is a common, serious and often devastating disorder resulting in significant morbidity, increased risk of mortality, and excessive healthcare costs as a consequence of complications which may occur as a result [2,13]. It remains one of the most challenging disorders encountered in medicine often requiring complex knowledge of pathophysiology across several organ systems and medical subspecialties. In the analysis of a patient with AKI, the physician is faced with determining etiology, pathophysiology, extent of renal injury, and reversibility whenever possible [1–3]. To this end, the AKIN, RIFLE, and KDIGO criteria have been proposed as the best estimates of renal injury [2,5,12]. These criteria do not establish pathophysiology or reversibility. Rather, the extent of renal injury is estimated by an absolute or relative rise in C, the duration of oliguria, or the need for RRT without signs of renal recovery. Neither GFR nor K is actually measured. Short-comings to these criteria include potential errors with C and dC/dt in estimating GFR due to dilution effects as well as differences in muscle mass and creatinine kinetics from patient to patient. Furthermore these estimates of renal injury are retrospective and have little impact on forecasting the actual extent of renal injury or the course of the azotemia during an episode of AKI [1,15,17,20,28]. Other methods for estimating the extent of renal injury have been proposed and considered possibly superior to RIFLE, AKIN, and KDIGO. This includes criteria proposed by Waikar and Bonventre (W&B) which use similar methods to AKIN and RIFLE with different metrics [16]. Although possibly superior in determining the extent of renal injury this method also falls short since GFR is not actually estimated. Other proposed methods include measuring K at short intervals to estimate GFR. Although promising it also falls short due to unpredictable changes in C related to dilution and the fact that the value for C is not in the steady state. Hence GFR is not actually measured by this method but again estimated based on values of C which are not always valid. Finally, measures of creatinine excretion have been studied to help estimate GFR. By these methods one estimates the excretion of creatinine with the understanding that excretion falls with the onset of injury and rises with time. By this method one attempts to determine the extent of renal impairment by the pattern of creatinine excretion. Again there are shortcomings in that creatinine excretion alone does not estimate actual GFR. A changing creatinine excretion does correlate with GFR but in a relative sense. One must know the baseline GFR to estimate the actual GFR and that is often not possible [14,15,17,18].

A real time, easy to perform, reliable and accurate estimate of GFR or K remains an elusive missing metric in AKI. Its usefulness in clinical medicine is clear in that one would have an accurate measure of renal injury, where the course and severity can be predicted in advance. Furthermore one could anticipate a plateau phase followed by a recovery which may also be anticipated well in advance of a changing C (S examples 1–8). A method to accurately estimate GFR or K, real time, is a welcome and overdue tool in the study of AKI.

As stated by the KDIGO guidelines for AKI, serum creatinine remains the best indicator of renal function and creatinine clearance should be measured whenever possible (2). The measurement of GFR in AKI can be accomplished by sophisticated methods using the injection of radioisotopes, which measure renal function in the non-steady state. Such methods are cumbersome, expensive, not readily available, nor practical in everyday use [34]. Methods to estimate creatinine clearance by serum C measurements are inaccurate and related to the volume effects on C as well as dC/dt, rendering these measurements prone to large error. Furthermore, these measurements require a distribution volume for creatinine which is also difficult to estimate and likewise prone to errors, rendering the serum creatinine based methods less than ideal for accurate estimates of K or GFR (S appendix).

The method described in this paper incorporates the logic behind creatinine mass balance where the ratio E/P determines the direction of the azotemia. From this, it follows that when E/P < 1, the patient is in AKI with the value for C rising. When E/P > 1, the patient is recovering from AKI with the value for C falling. Finally, when E = P, the patient is in a steady state, hence C is stable and K can be estimated or measured by the formula: K = U∗V/C. Furthermore, the absolute ratio E/P determines the extent of the renal injury. As noted under methods, a very low E/P is consistent with a large value for retained creatinine in the serum (An), a rising dC/dt, and a low K. Likewise, as E/P rises, An decreases, dC/dt falls, until one achieves a plateau where An = 0, E = P, and creatinine clearance can be estimated or measured directly. Finally, in the recovery phase, E/P > 1 and one sees a falling C (Fig. 1).

Taking this logic one step further, it is apparent that by taking the product E/P and Ks at any time t0, we incorporate into our calculation a value for baseline K, where upon the value of Ke is a reflection of Ks and directly affected by the ratio E/P. Hence, when E/P < 1, Ke < Ks. When E/P > 1, Ke > Ks. When E = P, Ke = Ks, with the actual value for Ke equal to Ks∗E/P. The significance of this method is highlighted by the following examples. In the case of two patients with a urine output of 500 ml in 24s and an identical estimation for P, the patient with the greater value for E will have a less severe renal injury for any level of Ks. In a similar way these same two patients with an E/P value of 0.5 will have very different values for Ke if in one case the Ks at t0 is 100 ml/min and in the other 40 ml/min where the value for Ke in these patients will be 50 ml/min and 20 ml/min respectively. One can appreciate that E/P determines the direction of the azotemia and Ks provides a baseline value used to determine Ke.

To test the accuracy of this hypothesis six different patient simulations were created, including variables of K at baseline and K after renal injury, body weight, age, and gender. These simulations were deemed to represent a variety of patient demographics representative of a hospital population with AKI. It was found that in each case Ke was similar to Ka with significant accuracy. It was also determined that 4 h time intervals were superior to 24 h time intervals with the difference between Ka and Ke usually less than 30% which equated to a difference of less than 5 ml/min. This time interval was deemed adequate to maintain a rather constant value for Ke. In that the graph of C as a function of time is not linear one would anticipate a longer timed urine collection would lead to higher values for E/P at the earliest time of renal injury (Fig. 1) leading to a Ke that exceeds Ka. The shorter time intervals allow for a more predictable and constant relationship between E/P and Ks throughout the course of AKI.

The same method was used to estimate K for patients with AKI seen in nephrology consultation by the author. The patients were divided into five groups with the predictive accuracy of the method measured in each group of patients. In those patients with E/P < 1, the predictive accuracy of the method was 85%, correlating with a rising C. Of the patients who did not meet the criteria, some of these were related to the onset of renal function recovery as determined by a subsequent falling C or rising E, which was often measured. In others not meeting criteria, the main reason was due to an over estimation for P. This led to a falsely low value for E/P and an underestimation of Ke highlighting the importance in the estimation for P. In those patients with an E/P > 1, 94% were associated with a falling C as expected, signifying renal function recovery. The majority of patients who did not meet criteria experienced an unexpected and abrupt loss of renal function signifying further renal injury in the face of critical illness and an unstable clinical situation. This was demonstrated as an abrupt drop in E when measured. The patients with an E/P = 1 had a predictive accuracy of 91% in forecasting a stable level of C. The majority of errors were encountered in those patients in whom P was over estimated leading to a falsely low value for E/P. In those patients with a Ke < 10 ml/min, 92% ultimately required RRT or achieved a peak level for C corresponding to a Ka < 10 ml/min by CKD-EPI. Of the patients who did not meet the criteria, the majority experienced renal function recovery manifested by falling C and rising E. Others underwent RRT with Ke > 10 ml/min for reasons such as hyperkalemia or pulmonary edema. Only three patients did not meet the criteria due to a low actual P and underestimation of Ke. Finally, of the 40 patients with AKI requiring dialysis who had a Ke measurement > 5 ml/min, all terminated RRT successfully manifested by a stable or falling C. This was often independent of urine volume per se, which was noted to increase appreciably several days before the onset of recovery was noted (S example 3).

Looking at the data further, it was noted that those patients with the lowest or highest values for E/P and Ke experienced the greatest change in C reflecting the extent of renal injury or recovery as one would expect (S examples 3, 4). Because GFR was not actually measured by radioisotope methods, the accuracy of comparing Ke to Ka could not be determined in this study.

In the study patients E was measured directly as the product of U∗V. Potential error could have been introduced by measuring a spot urine sample for U when the value may indeed have changed over time. By submitting the entire urine sample and then measuring U, this potential error would be overcome. Nevertheless, spot samples were used in each case in this study, introducing this possible error in E. Errors may be encountered in estimating P based on the fact that the level is indeed an estimate. Patients who are ill, or have prolonged hospital stays, experience a loss of muscle mass and a decrease in P, which is not always apparent. An estimate that P falls by 2% for each hospital day has been proposed [21,32]. This correction was made in this study. Based on observations of patients who were in hospital for long periods and who eventually achieved a steady state, actual measurements of P were significantly decreased indeed by as much as 50% in some patients (S examples 3, 5). This overestimate for P leads to a falsely low value for E/P and one would anticipate an underestimation of Ke. This was seen in some of the patients with a value of E/P < 1, which was associated with a stable or falling C, reflecting this error. Finally, the use of body weight was important in estimating P where lean body weight was used as opposed to actual body weight since the latter often over estimates P, especially in obese or edematous patients.

Another potential error seen in some study patients were those receiving large quantities of parenteral fluids leading to a dilution of C, and a falsely low dC/dt and Ks. This was reflected in several of the patients where Ke was quite low yet C remained stable or even fell, reflecting the dilution effect of parenteral fluids. In the majority of these patients, Ke was deemed to be accurate, in that over time, C eventually rose and in most of these patients the extent of the azotemia became obvious with RRT required in many cases (S example 2).

Interestingly errors in P would be expected to produce errors in the value for E/P which was indeed noted in several patients. On the other hand significant errors in estimation for P, or changes in V due to fluid administration, had little effect on Ke as shown in the patient simulations. The reason for this is based on the fact that there is a “self-correction” in the equation Ke = E/P × Ks, where an underestimation for P will lead to a falsely low E/P accompanied by a higher Ks. This is highlighted is simulated patient number 1, where a 20% fall in P estimation leads to a 2.9% fall in E and a 2.6% rise in Ks with the product of E/P and Ks relatively stable in that the changes occur in the opposite direction and cancel each other.

Finally, the accuracy of Ke required a value for Ks based on CKD-EPI measurements. Thus, the accuracy of Ks is directly related to the validity of CKD-EPI or any other formula used for GFR estimation [30–33,35–43]. The study also assumes there is no renal tubular secretion of creatinine, hence its contribution to false elevation of E could not be determined.

Despite these potential biases and errors introduced by the overestimation of P or the dilution of C, it became clinically apparent in the evaluation of the patients that these biases existed and by making adjustments in the estimate for P or the falsely low C, the predictive accuracy of the method was maintained and its validity overwhelmingly predictive in estimating K. The measure of Ke by the method described in this paper proved helpful in estimating the degree of renal injury in patients with AKI. During the course of their illness, one was able to observe a predictable pattern in AKI where Ke was able to forecast a course of worsening azotemia, the ultimate need for RRT days in advance, stabilization, recovery, or termination of RRT (S examples 1–8).

It is the opinion of the author that the method described in this paper improves on AKIN, RIFLE, KDIGO, as well as other methods proposed for estimating GFR in the non-steady state, and provides a very useful estimate of K in evaluating patients with AKI. In several of these patients, numerous measurements were taken which proved useful in quantifying the extent of the renal injury as well as tracking the course where a low Ke was associated with progressive azotemia, a rising Ke was associated with renal recovery and Ks = Ke was associated with a plateau phase (S examples 2,3,5,7). When one considers the fact that AKI can be accompanied by cumulative insults, or even rapid stabilization or recovery, the method provided in this paper allows one to follow these changes in Ke very closely and track the pattern of renal injury prospectively and with significant accuracy. The predictive powers of this method proved to be a useful clinical tool not available by any other method for renal function estimation present in the nephrology literature as far as one can tell.

Future studies should be undertaken to confirm the findings reported in this paper. Additional studies could also be undertaken to determine if a falling E/P over short time intervals is predictive of early renal injury such as in patients with sepsis or undergoing cardiothoracic surgery where early detection of AKI may allow earlier intervention. It may also prove interesting to correlate other markers of AKI to Ke to determine whether the predictive capacity of these markers, for showing tubular injury, is in fact enhanced [44–46]. Finally, the method should be confirmed for accuracy in studies where Ke is compared to actual GFR measurements by sophisticated radioisotope techniques. If this is confirmed, then the method described in this paper would prove to be a welcome addition to the metrics used for estimating renal injury in AKI.

## 5. Conclusion

The method described in this paper provides an accurate and easy to perform tool for estimating K in the patient with AKI. When E/P is less than 1 the patient is in AKI with C rising. When E/P is greater than one the patient is in a recovery pattern with C falling. The ratio of E/P reflects the severity of azotemia and the size of dC/dt. When E is equal to P the patient is in a steady state and K can be estimated or measured directly. Multiplying E/P by Ks at any time 0 provides a very good estimate of actual K, real time, in the patient with AKI.

## Supplementary Material

1

## Figures and Tables

**Table 1:** Simulated patient 1. 24 year male, wt 63 kg, P 1440 mg/day, K falls from 100 ml/min to 10 ml/min. The bold and italics simply serve to highlight certain text and values.

C	E	E/P	Ks	E/P*Ks
24 h intervals				
1	361	0.25	100	25
3.84	699	0.485	26	12.63
5.79	935	0.65	17.27	11.23
7.12	1094	0.76	14	10.67
8.03	1201	0.83	12.45	10.34
8.66	1280	0.89	11.55	10.28
9.08	1345	0.934	11.01	10.29
9.37	1364	0.947	10.67	10.11
9.57				
4 h intervals				
1	35	0.146	100	14.6
1.54	46	0.192	65	12.5
2.05	58	0.242	49	11.8
2.53	61	0.254	39.5	10
3	88	0.367	29.4	10.8
3.4	88	0.367	26.3	9.7
3.8				
9.08	217	0.9	10.67	9.6
9.14				
4 h intervals				
1	34	0.146	100	14.6
1.5	44	0.183	66.7	12.2
1.97	53	0.221	50.8	11.2
2.41	63	0.263	41.5	10.9
2.82	72	0.3	35.5	10.7
3.2	82	0.342	31.25	10.7
3.55				

Assume 20% fall in P to 1152 mg and fall in V from 60% to 50% BW.

**C** = Creatinine concentration—in mg/dl.

**E** = Creatinine excretion—in mg.

**Ks** = Static GFR.

**E/PxKS** = Ke = Estimated GFR.

**Table 2:** Simulated patient 2. 24 year male, wt 63 kg, P 1440 mg, K falls from 100 ml/min to 50 ml/min. The bold and italics simply serve to highlight certain text and values.

C	E	E/P	Ks	E/P*KS
24 h intervals				
1	1119	0.78	100	78
1.85	1390	0.965	54	52.1
1.98				
4 h intervals				
1	137	0.57	100	57
1.271	164	0.683	78.7	53.7
1.47	191	0.795	68	54.1
1.61	198	0.825	62.1	51.24
1.72	210	0.875	58	50.9
1.8	220	0.916	55.6	50.9
1.852				
4 h intervals				
1	129	0.54	100	54
1.2	148	0.62	83.3	51.65
1.34	163	0.68	74.6	50.73
1.43	173	0.72	70	50.4
1.49	179	0.75	67	50.25
1.53	186	0.78	65.4	51
1.55				

Assume 20% fall in P to 1152 mg and fall in V from 60% to 50% BW.

**C** = Creatinine concentration—in mg/dl.

E = Creatinine excretion—in mg.

**Ks** = Static GFR.

**E/PxKS** = Ke = Estimated GFR.

**Table 3:** Simulated patient 3. 70 year female, wt 55 kg, P 701 mg, K falls from 81.2 ml/min to 34.8 ml/min, V = 0.6 wt = 33 L. The bold and italics simply serve to highlight certain text and values.

C	E	E/P	Ks	E/P*Ks
24 h intervals				
0.6	494	0.7	81.2	56.8
1.225	688	0.98	39.73	38.94
1.263	691	0.985	38.54	37.96
1.293	693	0.988	37.65	37.2
1.316	695	0.99	37	36.6
1.334	697	0.99	36.5	36.14
1.345				
4 h intervals				
0.6	58	0.49	81.2	40
0.78	71	0.6	62.4	37.8
0.92	81	0.69	52.91	36.5
1.03	90	0.766	47.26	36.2
1.113				
4 h intervals				
0.6	55	0.47	81.2	38.2
0.74	66	0.56	65.8	36.8
0.84	75	0.64	57.96	37.1
0.91	79	0.68	53.5	36.4
0.965				

Assume 20% fall in P to 561 mg and fall in V from 60% to 50% BW.

**C** = Creatinine concentration—in mg/dl.

**E** = Creatinine excretion—in mg.

**Ks** = Static GFR.

**E/PxKS** = Ke = Estimated GFR.

**Table 4:** Simulated patient 4. 70 year female, wt 60 kg, P 766 mg, K falls from 38 ml/min to 10 ml/min, V = 0.6 × 60 = 36 L. The bold and italics simply serve to highlight certain text and values.

C	E	E/P	Ks	E/P*Ks
24 h intervals				
1.4	302	0.39	38	14.82
2.69	453	0.59	19.8	11.7
3.56	557	0.73	14.9	10.9
4.14	626	0.82	12.8	10.5
4.53	672	0.88	11.7	10.3
4.79	705	0.92	11.1	10.2
4.96				
4 h intervals				
1.4	38	0.297	38	11.29
1.65	42	0.328	32.24	10.57
1.89	49	0.383	28.15	10.78
2.11				
4 h intervals				
1.4	36	0.28	38	10.64
1.62	42	0.33	32.83	10.83
1.82	45	0.35	29.2	10.22
2.01				

Assume 20% fall in P to 613 mg and fall in V from 60% to 50% BW.

**C** = Creatinine concentration—mg/dl.

**E** = Creatinine excretion—in mg.

**Ks** = Static GFR.

**E/PxKS** = Ke = Estimated GFR.

**Table 5:** Simulated patient 5. 60 year male. wt 60 kg, P 1015 mg, K falls from 33.6 ml/min to 5.1 ml/min, V = 36 L. The bold and italics simply serve to highlight certain text and values.

C	E	E/P	Ks	E/P*Ks
24 h intervals				
2.1	234	0.231	33.6	7.76
4.27	378	0.372	16.51	6.14
6.04	497	0.49	11.67	5.72
7.48	594	0.585	9.42	5.51
8.65	669	0.66	8.15	5.38
9.61	734	0.723	7.33	5.3
10.39	788	0.776	6.78	5.26
11.02	828	0.816	6.4	5.22
11.54				
4 h intervals				
2.1	29	0.172	33.6	5.78
2.49	36	0.213	28.9	6.16
2.86	39	0.231	25.45	5.88
3.22				
4 h intervals				
2.1	27	0.16	33.6	5.38
2.46	33	0.195	28.7	5.6
2.8	36	0.213	25.2	5.37
3.13				

Assume 20% fall in P to 812 and fall in V from 60% to 50%.

**C** = Creatinine concentration—in mg/dl.

**E** = Creatinine excretion—in mg.

**Ks** = Static GFR.

**E/PxKS** = Ke = Estimated GFR.

**Table 6:** Simulated patient 6. 60 year male, wt 80 kg, P 1330 mg, K falls from 77 ml/min to 25 ml/min. The bold and italics simply serve to highlight certain text and values.

C	E	E/P	Ks	E/P*Ks
24 h intervals				
1.2	696	0.523	77	40.3
2.52	1032	0.76	36.7	27.9
3.14	1186	0.89	29.4	26.2
3.44	1263	0.95	26.8	25.5
3.58	1301	0.98	25.8	25.3
3.64				
4 h intervals				
1.2	78	0.35	77	27
1.5	97	0.44	61.6	27.1
1.76	116	0.5	52.5	26.3
1.97				
4 h intervals				
1.2	81	0.36	77	27.7
1.44	101	0.45	64.1	28.8
1.65	105	0.47	56	26.3
1.83				

Assume 20% fall in P to 1064 mg and fall on V from 60% to 50%.

**C** = Creatinine concentration—in mg/dl.

**E** = Creatinine excretion—in mg.

**Ks** = Static GFR.

**E/PxKS** = Ke = Estimated GFR.

## Abbreviations:

AKI: acute kidney injury
GFR: glomerular filtration rate
K: creatinine clearance
Ka: actual K
Ke: estimated K
Ks: static K
C: serum creatinine concentration
U: urine creatinine concentration
E: creatinine excretion
P: creatinine production
Vd: creatinine distribution volume
