# Supplementary material for: The description of a method for accurately estimating creatinine clearance in acute kidney injury
Source: Math Biosci. Author manuscript; Available in PMC 2021 May 6. (PMC8101066; doi:10.1016/j.mbs.2016.02.010)

**SUPPLEMENTARY MATERIAL**

Appendix: The three formulas for measuring K using serum C in the non- steady state.

Examples 1 through 8: Patient Examples are included for clarification on using the method.

**APPENDIX**

The three formulas for measuring K using serum C in the non- steady states are based on creatinine mass balance and are as follows:

1. Vd * (C2 – C1)/T = P – (C2 – C1/2) * K/100 * 1440 (Ref 27,28)
2. K = P/C – Vd * [dC/dt /C] (Ref 29)
3. K = C * Ks/C(mean) * [1 – (24*ΔC/Δt * Max Δ C/Day)] (Ref 30)

Where Vd = creatinine distribution volume and P is estimated by the standard formula under the methods section.

The formulas assume a single compartment model for C. Additionally; their accuracy depends on valid values for Vd, C, dC/dt, and ΔC, which are often not met as explained in the manuscript.


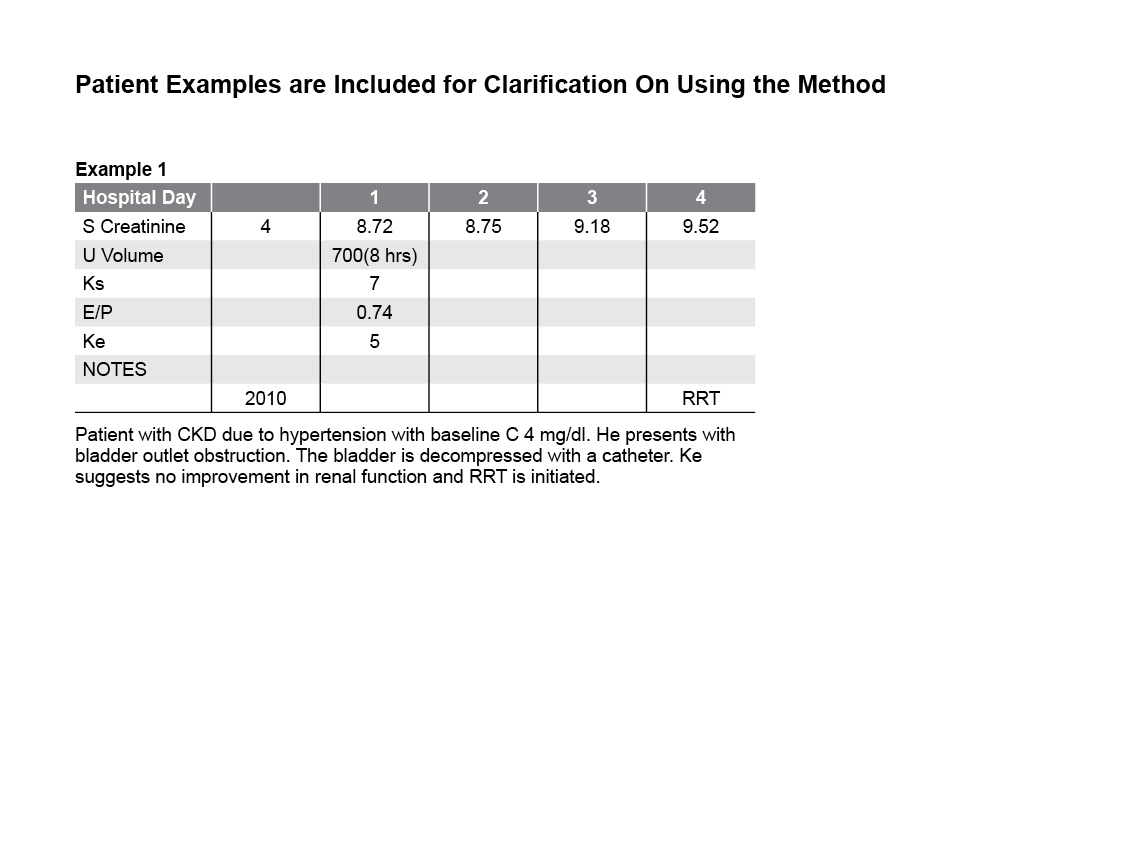


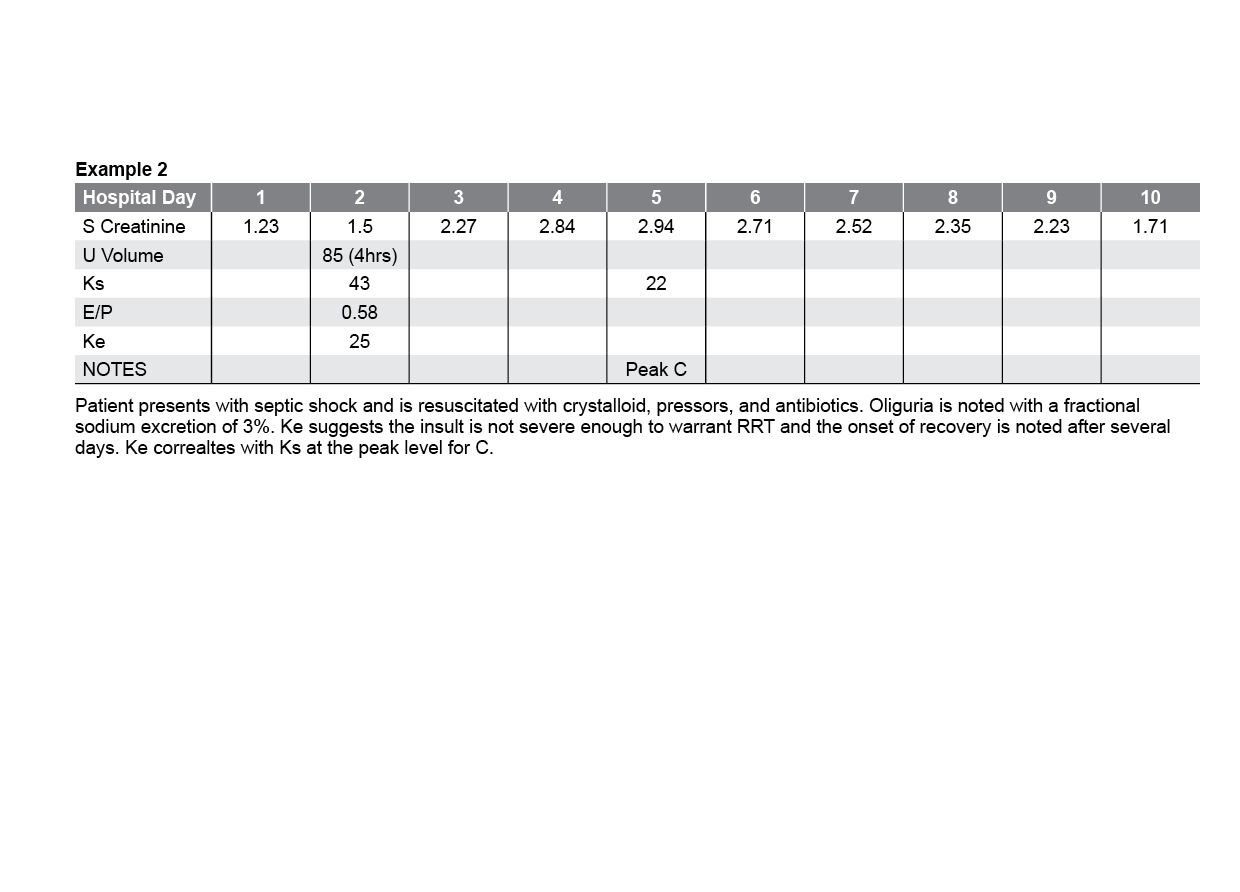


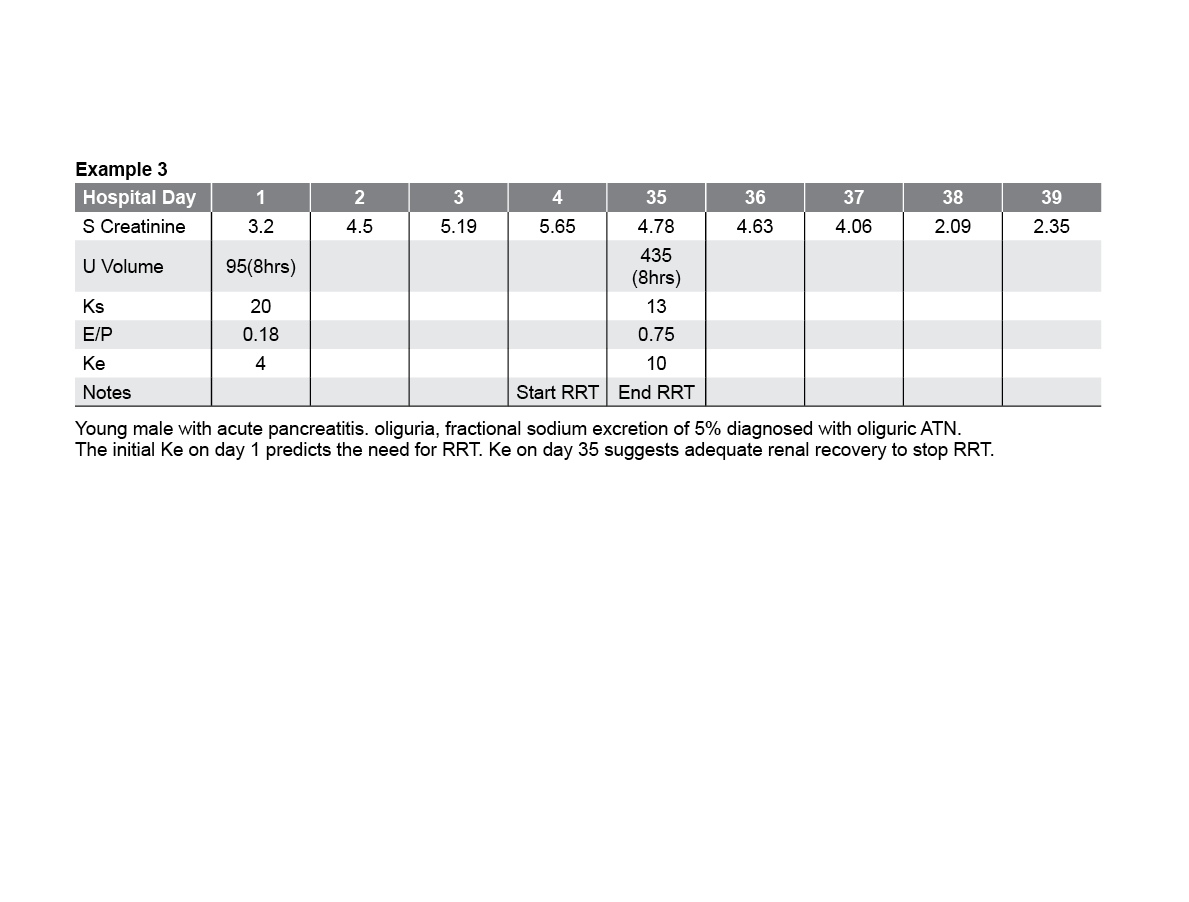


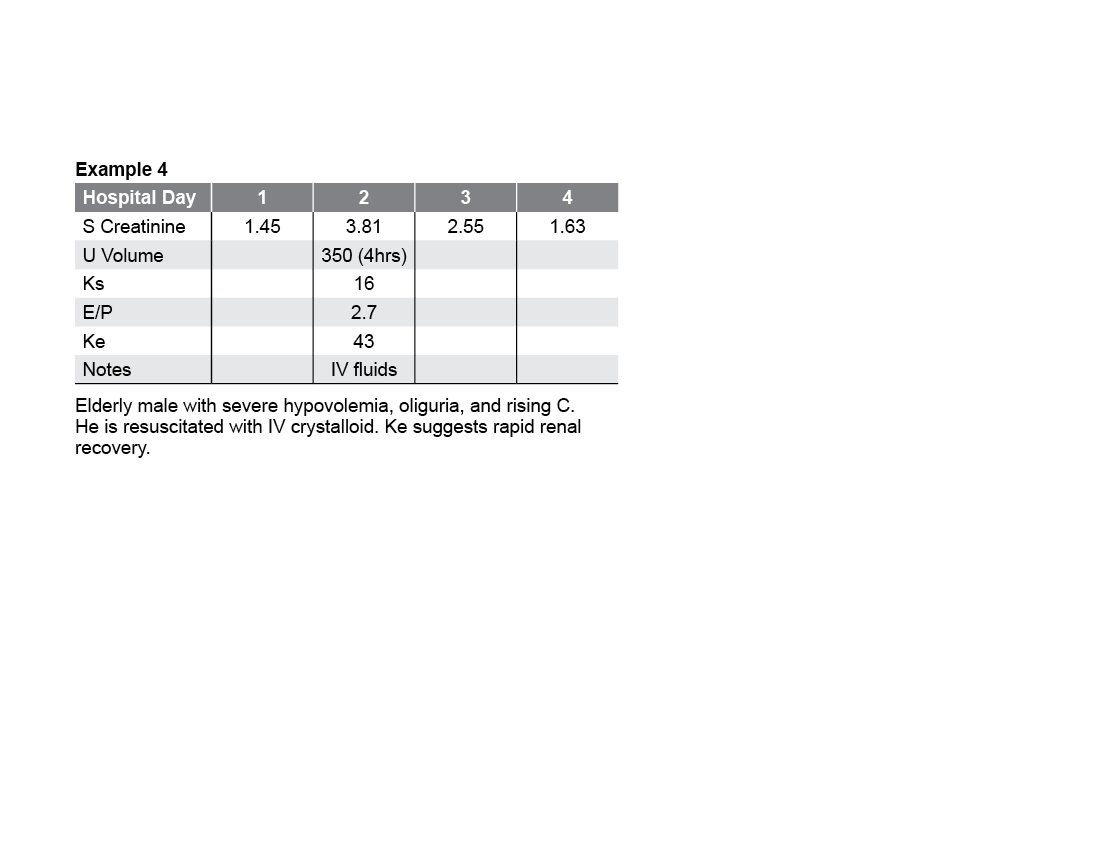


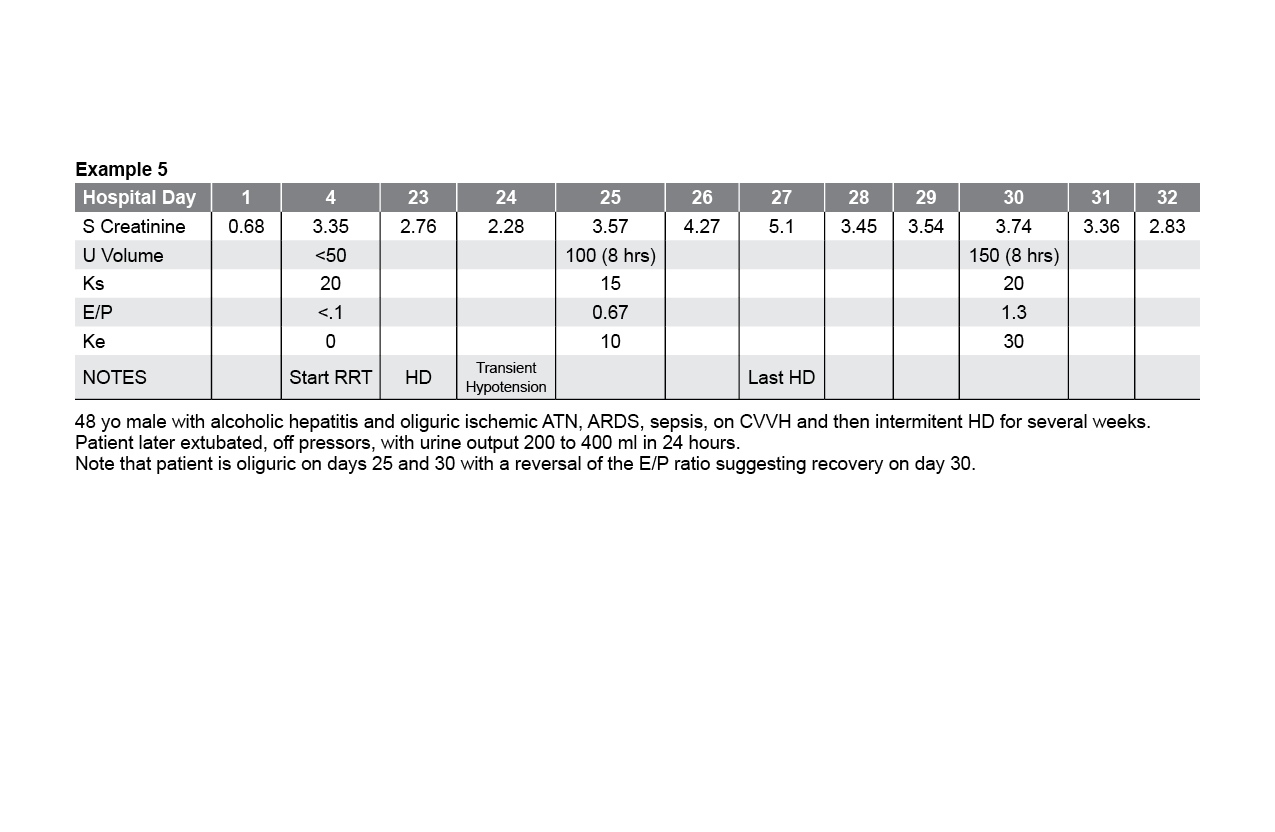


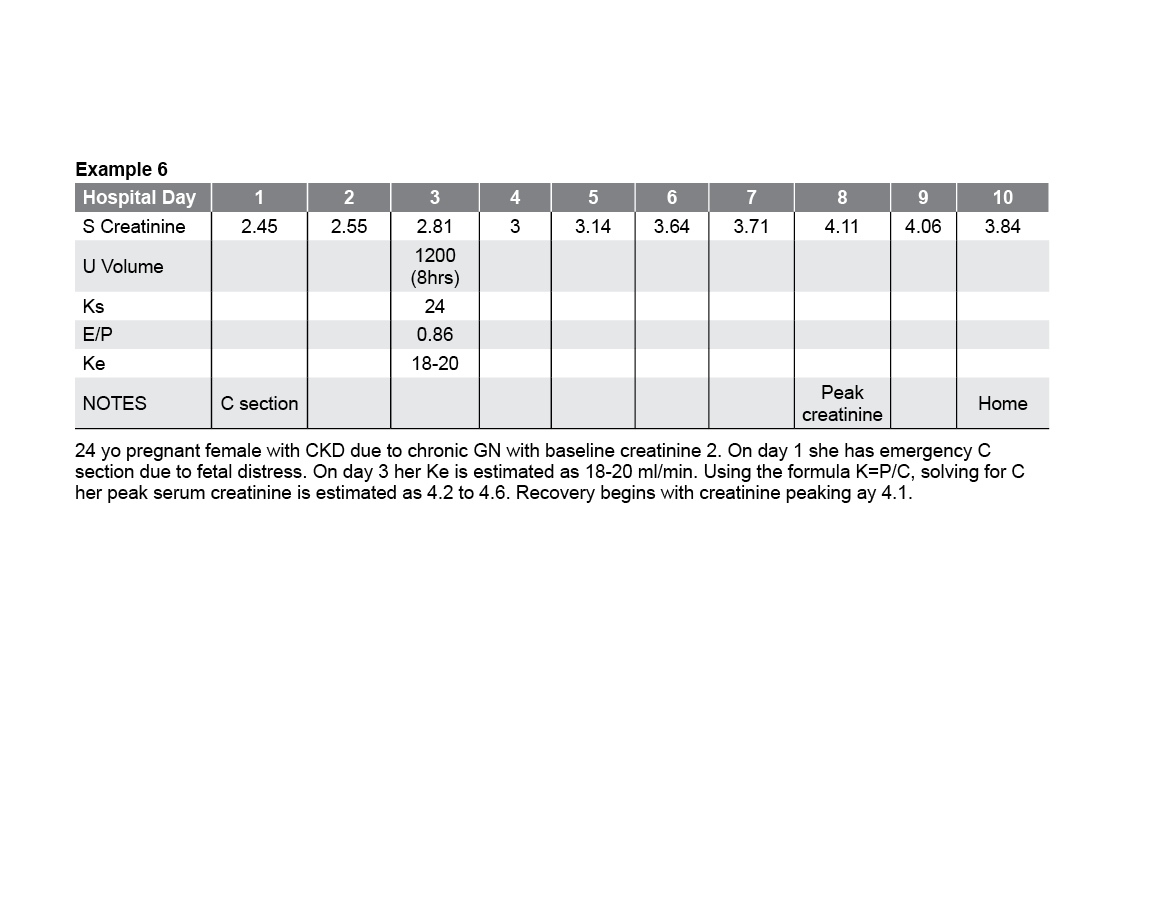


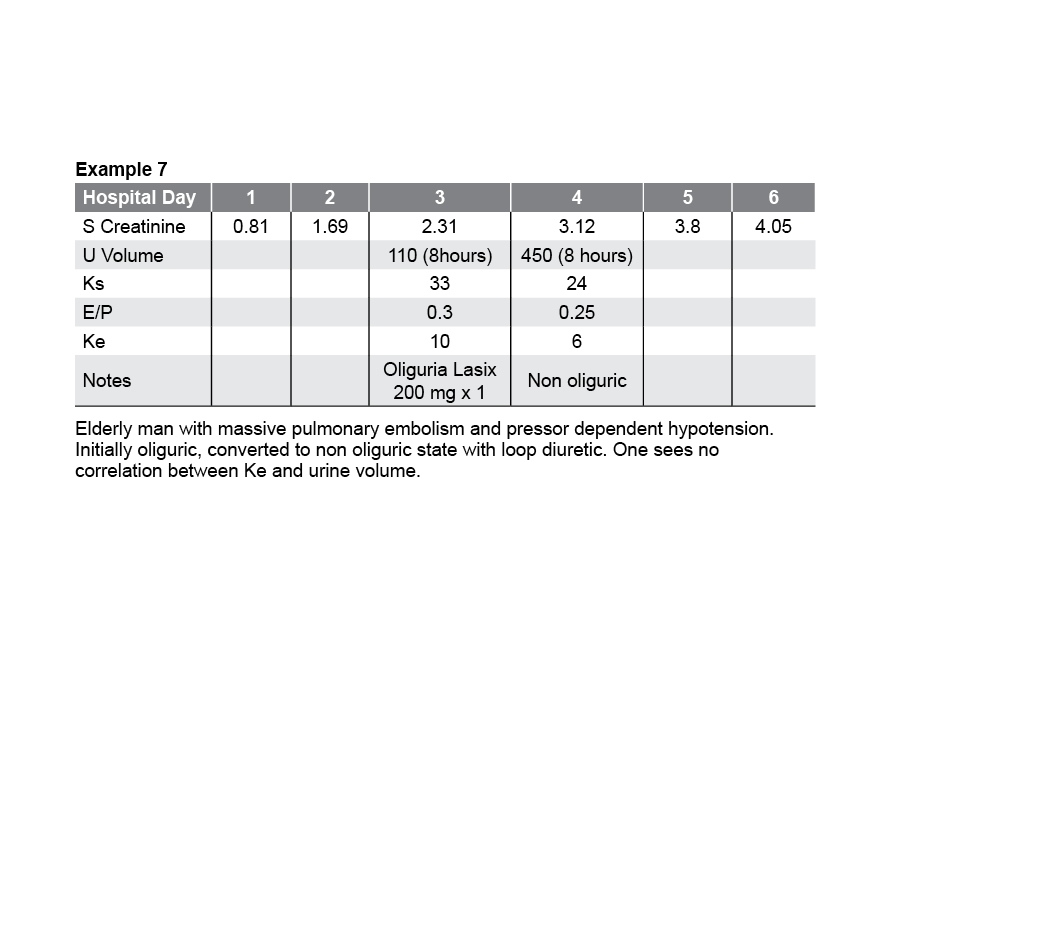


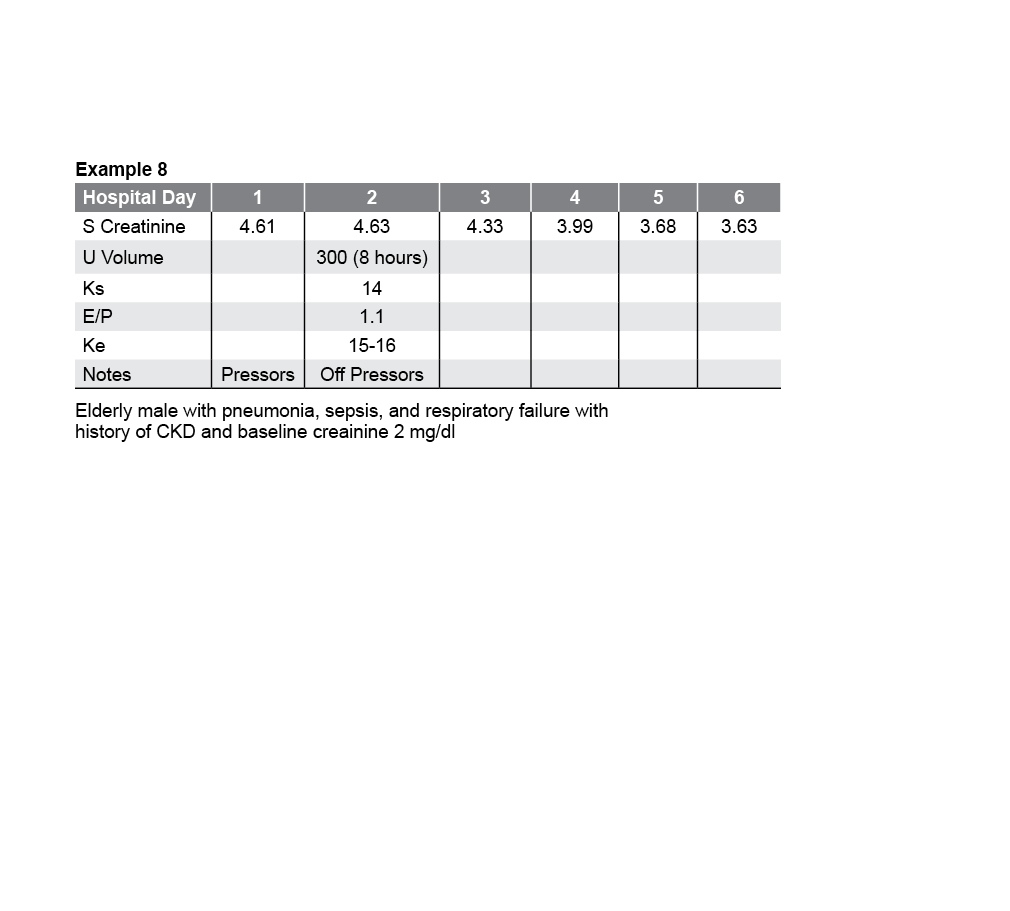

Supplement: 1 [file NIHMS817929-supplement-1.docx]
